# The efficacy of injectable bone fillers for alveolar ridge preservation: a microcomputed tomographic analysis

**DOI:** 10.1186/s40729-026-00673-7

**Published:** 2026-03-30

**Authors:** A. Ramanauskaite, S. Kallab, M. Agirman, P. Parvini, K. Obreja, J. Lorenz, F. Schwarz, K. Becker

**Affiliations:** 1 Department of Oral Surgery, Implantology and Oral Medicine, Goethe University ZZMK Carolinum, Frankfurt, Germany; 2 Department of Orthodontics, Universitätsklinikum Düsseldorf, Düsseldorf, Germany; 3 Department for Oral, Cranio-Maxillofacial and Facial Plastic Surgery, Goethe University University Hospital Frankfurt, Frankfurt, Germany

**Keywords:** Preclinical study, Alveolar ridge preservation, Histological analysis

## Abstract

**Aim:**

To evaluate the efficacy of injectable bone fillers for alveolar ridge preservation (ARP).

**Materials and methods:**

Mandibular premolars (P2, P3, P4) were bilaterally extracted in nine beagle dogs. Each tooth underwent hemisection, with the mesial root devitalized and filled with calcium hydroxide, while the distal roots were extracted. This resulted in six sockets per dog, which were randomly assigned to four injectable test materials (T1–T4), one control (C), and one negative control group (N). Primary wound closure was achieved in all groups except for N. After 12 weeks, tissue blocks were analyzed using micro-computed tomography (micro-CT). The primary outcome was bone volume fraction (BV/TV, %). Secondary outcomes included trabecular thickness (Tb.Th, mm), trabecular separation (Tb.Sp, mm), bone surface to bone volume ratio (BS/BV, mm^2^/mm^3^), buccal bone defect volume (BBD, mm^3^), vertical bone height (VBH, mm), buccal wall thickness (BBW, mm) and lingual wall thickness (LBW, mm). Data were analyzed using the Kruskal–Wallis test.

**Results:**

After 12 weeks of healing, all groups were associated with a similar BC/TV values (64.6%, 68.2%, 69.0%, 66.5%, 76.5% and 79.8% in the T1, T2, T3, T4, C and N groups, respectively; p > 0.05 for all between group comparisons). No statistically significant differences were found among groups for Tb.Th, Tb.Sp, BS/BV, BBD, VBH, BBW and LBW.

**Conclusions:**

Within its limitations, the present study showed comparable efficacy of injectable bone fillers in maintaining alveolar ridge dimensions compared with the C and N groups.

**Clinical relevance:**

Injectable bone fillers represent a convenient and potentially effective alternative for alveolar ridge preservation procedures.

## Introduction

Tooth loss triggers a cascade of bone remodeling processes that result in significant dimensional changes of the alveolar ridge [1–3]. Human re-entry studies demonstarted that the remarkable dimentional alterations occurs within the first 3 to 6 months resulting in horizontal bone loss of 29–63% and vertical bone loss of 11–22% [1]. This marked volumetric decrease in alveolar ridge can promise future dental implant placement and aesthetic outcomes, highlighting the need for effective alveolar ridge preservation (ARP) strategies [4, 5].

The ARP is aimed at attenuating post-extraction dimensional changes following tooth extraction, thereby maintaining the structural integrity of the alveolar ridge for implant placement [6]. Different ARP techniques, which typically involve use of different bone graft materials, barrier membranes, or biologics, have been demonstrated to reduce post-extraction bone loss compared to unassisted alveolar socket healing [7–10]. One systematic review that included solely randomized controlled trials (RCTs) revealed that compared to spontaneous healing ARP significnatly reduces horizontal (mean difference (MD) = 1.99 mm; 95% CI 1.54–2.44), vertical mid-buccal (MD = 1.72 mm; 95% CI 0.96–2.48) and vertical mid-lingual (MD = 1.16 mm; 95% CI 0.81–1.52) bone resorption [7]. Although the most effective ARP protocol could not be idientifed, in preventing horizontal bone resorption, a larger ARP effect was found in patients treated with particulated xenogenic and allogenic bone substitutes covered by an resorbable collagen barrier [7].

Regarding the clinical handling of the particulated bone fillers, previous experimental studies have shown that the compressive forces applied to particulated bone substitute during the ARP directly affects defect fill in the apical region of the extraction socket, potentially compromising new bone formation [11, 12]. Subsequently, it might be speculated that the outcomes of ARP could be further enhanced by using injectable and flowable bone fillers, which offer easier clinical handling independent of application pressure, subsequently leading to more uniform grafting across all compartments of the extraction socket. Therefore, the aim of this preclinical study was to assess the efficacy of four novel experimental injectable bone fillers in preserving alveolar ridge dimensions when applied to fresh extraction sockets, using microcomputed tomography (micro-CT) in an established canine model.

## Materials and methods

### Animals

The methodology was previously reported in a publication presenting the histomorphometic outcomes if this study [13]. In brief, nine male beagle dogs (Breed: HsdRcc:DOBE, Marshall, France), each at least 12 months old and weighing between 9.8 and 13.4 kg, were included in the study. Animal housing, care, and environmental conditions complied with European regulations (Directive EU/2010/63). The dogs were housed under controlled laboratory conditions (temperature 15–21 °C, 12 h light/dark cycle, daily monitoring of humidity and temperature). After an acclimatization period of at least 15 days, the experiments were conducted. To minimize complications, animals were kept individually during the postoperative healing phase. A commercially available soft diet (SAFE, France) was provided twice daily before and after surgery, and fresh drinking water was available ad libitum. Feed and water were free of contaminants that could have negatively influenced the study outcome.

All surgical procedures were performed at NAMSA (Chasse-sur-Rhône, France), a facility accredited by AAALAC International and registered with the French Department of Agriculture. The study protocol (APAFIS#12,878–201,801,021,136,883) received approval from the NAMSA Ethics Committee and followed the 3R principles (Replace, Reduce, Refine) in animal research. The study adhered to the ARRIVE Guidelines 2.0 for comprehensive and transparent reporting (Percie du Sert et al., 2020).

### Study design

**Fig. 1:**
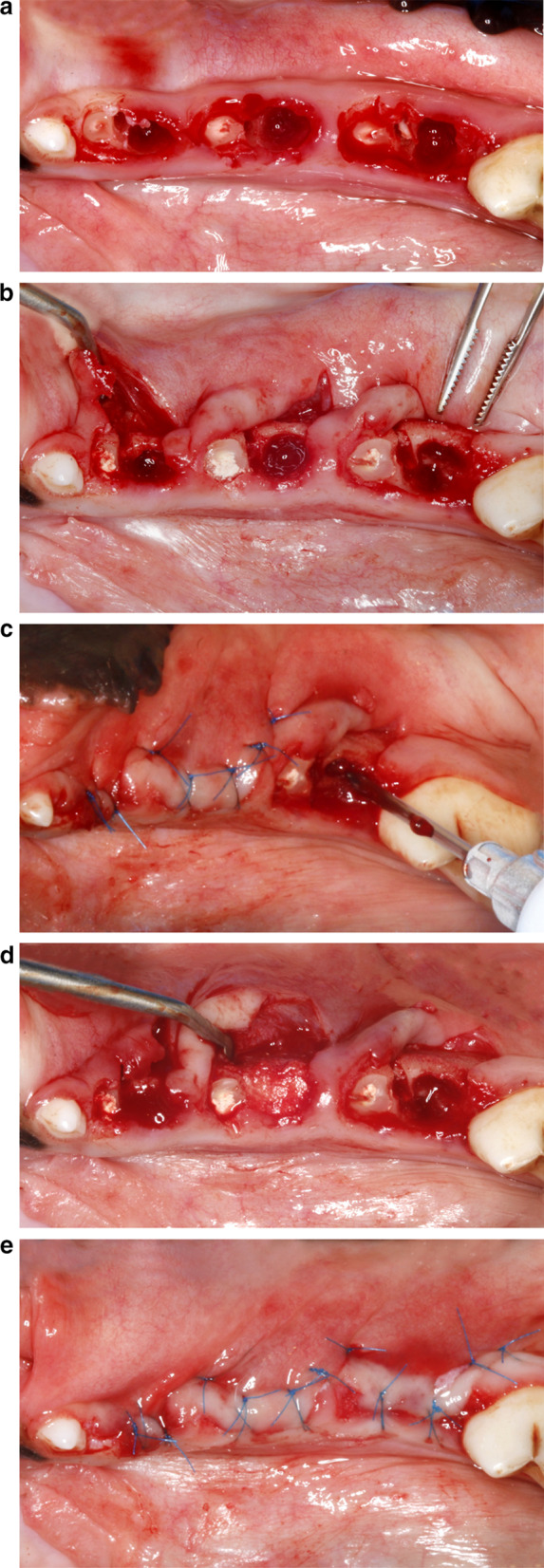
Surgical procedures. **a** Situation following careful hemisection under preservation of the interradicular bone. **b** The distal root was removed and the mesial root prepared for a provisional endodontic treatment (i.e. pulp extirpation, root canal enlargement, filling using calcium hydroxide paste, sealing using a self-curing glass-ionomer cement material). **c** The distal alveolar socket was completely filled by loosely applying the respective test and **d** control materials. Negative control sites were left untreated and ensured to be filled with blood. **e** A tension-free wound closure at the experimental sites was obtained by means of buccally elevated mucosal flaps

In all dogs, the mandibular premolars (P2, P3, P4) were bilaterally assigned to either ARP or spontaneous healing (Fig. 1). After hemisection and tooth extraction, the six resulting sockets in each animal were randomly assigned to one of four experimental test materials, a control material (Geistlich, Wolhusen, Switzerland), or a negative control group (N) using a computer-generated randomization code.

The test groups were categorized based on the type of injectable bone filler material used:

- Test 1 (T1): Lyophilized bovine bone particles (0.12–0.18 mm) combined with porcine collagen (M2L, Geistlich), reconstituted with autologous blood.
- Test 2 (T2): Lyophilized bovine bone particles (0.12–0.18 mm) combined with porcine collagen (M2L, Geistlich), reconstituted with saliva.
- Test 3 (T3): A pre-hydrated material containing bovine bone particles (0.12–0.18 mm) and porcine collagen (M2rtu, Geistlich).
- Test 4 (T4): Lyophilized bovine bone particles (0.12–0.18 mm) combined with porcine collagen (M4L, Geistlich), reconstituted with saliva.
- Control (C): Particulated xenogeneic bone substitute material (Bio-Oss®, Geistlich).
- Negative Control (N): Spontaneous healing of the extraction socket without augmentation.

Each treatment, including test and control groups, was applied to six extraction sockets across all animals (n = 9).

### Pre-operative procedure and anesthesia protocol

All dogs underwent a full-mouth scaling within two days prior to surgery. To prevent infection, spiramycin and metronidazole (Stomorgyl®, Merial) were administered orally once daily for two days before the procedure.

On the day of surgery, premedication was given via intravenous injection of medetomidine (Dorbene Vet®, Zoetis, NJ, USA) and buprenorphine (Buprecare®, Axience, Pantin, France). Anesthesia was then induced with an intravenous injection of ketamine (Ketamine® 1000, Virbac, Carros, France). Once anesthetized, each dog was intubated, placed on mechanical ventilation, and maintained under general anesthesia with isoflurane (IsoFlo®, Zoetis). Throughout the procedure, intravenous fluids (Ringer lactate, Baxter, Deerfield, IL, USA) were administered to maintain hydration. Additionally, a pre-operative subcutaneous dose of carprofen (Rimadyl®, Zoetis) was given as an anti-inflammatory measure.

To prevent corneal drying, a neutral ophthalmic ointment (Ocrygel®, TVM, Berlin, Germany) was applied to both eyes and reapplied as necessary. Lidocaine with adrenaline (Aguettant, Langenfeld, Germany) was injected bilaterally at the surgical sites, with the dosage determined at the surgeon’s discretion. Each dog was positioned laterally on a heated pad during surgery. A rectal probe was placed to monitor body temperature. Additionally, electrocardiogram readings, peripheral noninvasive arterial blood pressure, and oxygen saturation levels were continuously monitored.

### Sample size calculation

Determining the required sample size was not possible due to a lack of sufficient reference data in the literature regarding these ARP protocols.

### Surgical procedures

Circumferential marginal incisions were made around each mandibular premolar (P2, P3, P4). A bucco-lingual hemisection was performed using a fissure bur under saline irrigation, leaving the interradicular crestal bone intact. The distal root was then mobilized with a syndesmotome and a luxator, and subsequently extracted with controlled apical and rotational movements using elevators and forceps. The socket was irrigated with sterile saline to remove debris, and any remaining root fragments that could not be extracted were documented.

The mesial root was decoronated at the cemento-enamel junction. If necessary, the pulp chamber was accessed using a bur, and the pulp tissue was extirpated with a barbed broach or Hedström file. The root canal was prepared up to ISO size 25 with saline irrigation, dried with paper points, filled with calcium hydroxide paste, and coronally sealed with a glass-ionomer cement. The respective test and control materials were placed into the distal extraction sockets, ensuring complete but not excessive filling up to the crestal bone level. In the negative control sites, the sockets were left empty to allow natural blood clot formation. To achieve tension-free primary closure, buccal mucoperiosteal flaps were elevated and, when required, a vertical releasing incision was performed. Finally, the maxillary premolars (P2, P3, P4) were shortened coronally without pulp exposure to avoid traumatic occlusion.

### Postoperative care

Following surgery, each dog was transferred to a recovery area, where it was monitored until it regained sternal recumbency. Once stable, the animals were returned to their enclosures and closely observed for overall health. Postoperative antibiotic prophylaxis with spiramycin and metronidazole was administered orally once daily for 14 days. Carprofen (Carprodyl®, Ceva) was given once per day for six days to manage inflammation.

Pain management included a subcutaneous injection of buprenorphine (Buprecare®, Axience) at the end of surgery, followed by twice-daily administration for the next two days. Local disinfection was performed daily using a chlorhexidine solution until the sutures were removed approximately two weeks postoperatively. Postoperative health was assessed daily, including evaluation of wound healing (redness, swelling, dehiscence, or signs of infection), and body weight was recorded biweekly.

### Specimen retrieval

The animals were euthanized 12 weeks after surgery. For this procedure, anesthesia was induced using an intramuscular injection of tiletamine-zolazepam (Zoletil®, Virbac), followed by a subcutaneous dose of buprenorphine (Buprecare®, Axience). After intubation, the dogs were placed on mechanical ventilation and maintained under general anesthesia with isoflurane (IsoFlo®, Zoetis). Catheters were introduced into the carotid arteries, and all animals were administered heparin (300 IU/kg, intravenous) (Heparin Choay, Sanofi-Aventis, Paris, France) to prevent clotting.

Euthanasia was carried out via an intravenous injection of a lethal dose of pentobarbital (Dolethal®, Vetoquinol). Subsequently, specimens were fixed in 10% neutral buffered formalin, dehydrated in ascending ethanol concentrations, cleared in xylene, and embedded in polymethyl methacrylate for undecalcified sectioning. Micro-CT scans of each mandibular half were performed prior to histological processing.

### Image processing and analysis of the bone micromorphometry

In total, 54 extraction sockets were created (9 animals × 6 sockets each). Due to sample loss and technical exclusions (e.g., inadequate embedding, residual root fragments, undefined defect formation, or insufficient defect height for standardized analysis), 46 specimens were available for micro-CT analysis. All samples were scanned using a cone beam micro-computed tomography system (µCT 40, SCANCO Medical, Switzerland) at 70 kVp, 114 µA, and 786 ms integration time. Images were reconstructed at a nominal isotropic voxel size of 16.1 µm and analyzed using the manufacturer’s evaluation software (Scanco Medical). A Gaussian filter was applied for noise reduction and edge enhancement. The datasets were aligned to the long axis of the mesial root.

For the definition of the volume of interest (VOI), a standardized rectangular region (100 px width × 200 px depth) was placed at the buccal aspect of the mesial root. The apical extent of the VOI was defined as the most apical level of the graft material, while the crestal bone level served as the coronal reference. In cases where the VOI exceeded the mandibular bone contour, it was individually adjusted to remain within the cortical boundaries.

**Fig. 2:**
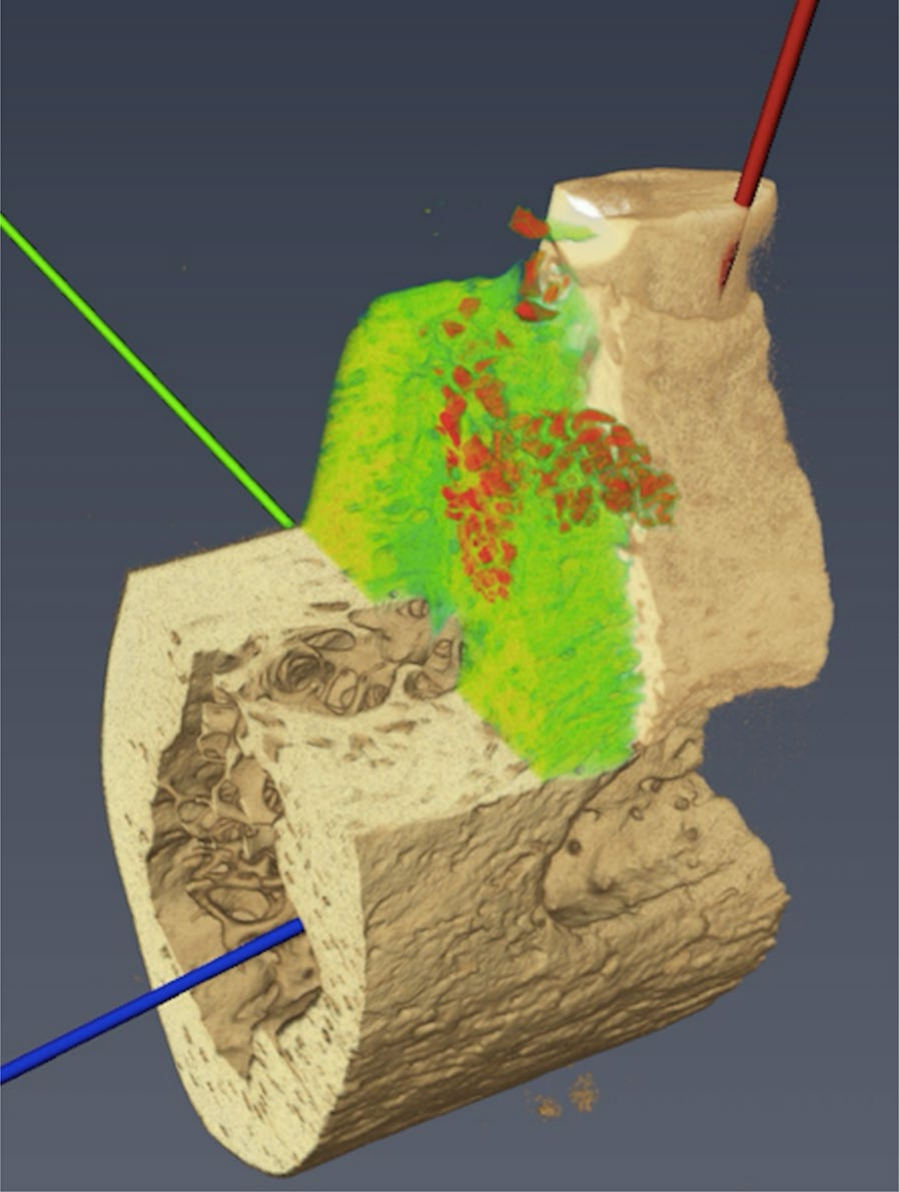
Illustration of the volume of interest (VOI) selection. Micro-CT dataset after axis alignment (red: Z-axis/ vertical axis, blue: X-axis/ mesial-distal, green: Y-axis/ facial-lingual). The green part represents the vertical extension of the augmentation that extended ot the apex of the mesial root, while the red area indicates the graft material visible in the Micro-CT scan

**Fig. 3:**
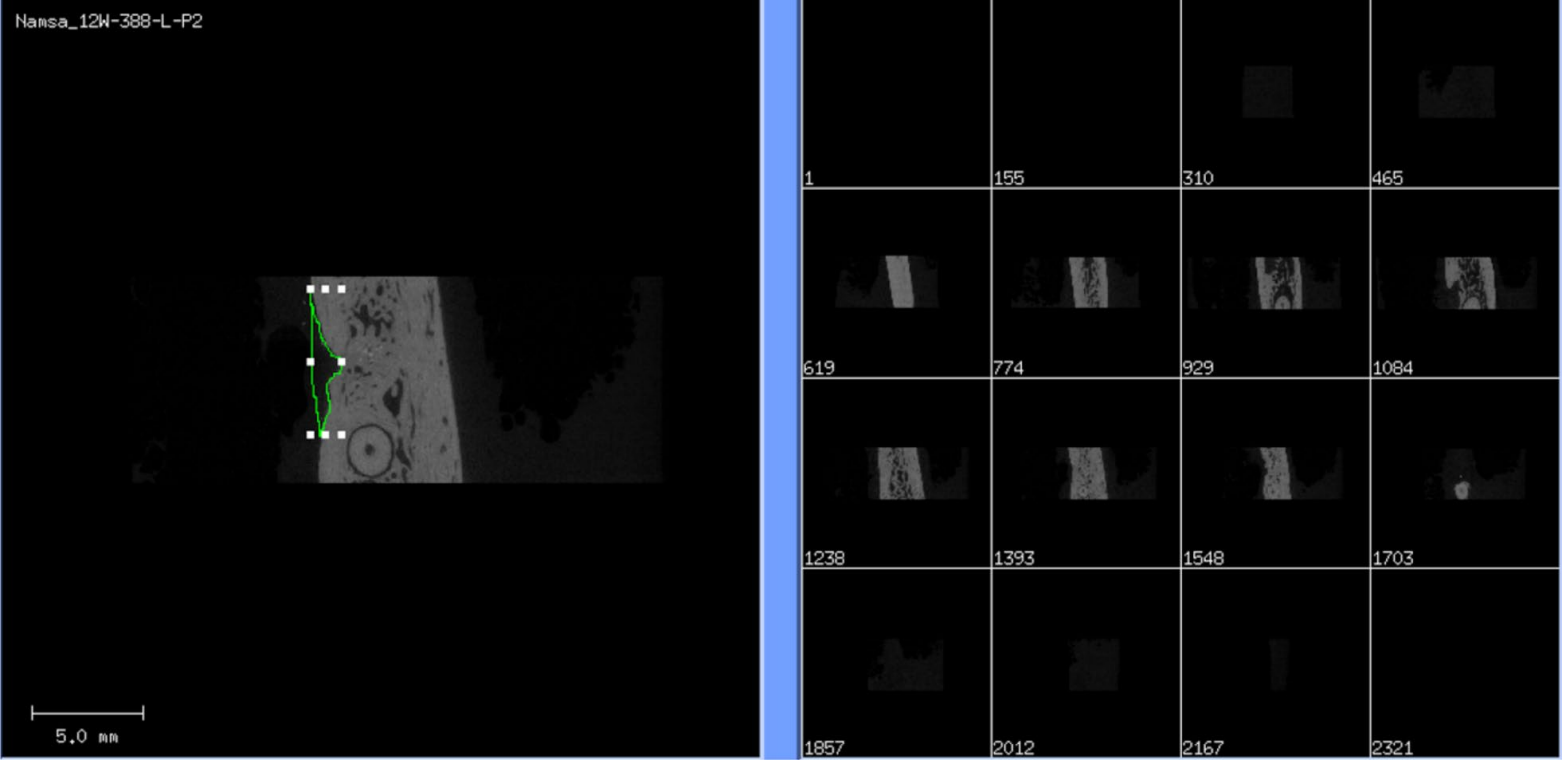
Illustration of the buccal bone defect (BBD, mm^3^) marking

The following volumetric parameters were quantified within the VOI (Fig. 2):

- Bone volume fraction (BV/TV, %): ratio of bone volume to total VOI (i.e., the relative proportion of bone within the VOI; primary outcome);
- Trabecular thickness (Tb.Th, mm): average thickness of trabeculae (i.e., mean width of the trabecular structures);
- Trabecular separation (Tb.Sp, mm): mean distance between trabeculae (i.e., the average spacing between trabecular structures);
- Bone surface to bone volume ratio (BS/BV, mm^2^/mm^3^): ratio of trabecular surface area to trabecular volume, calculated automatically by the manufacturer’s software within the VOI, reflecting structural complexity;
- Buccal bone defect volume (BBD, mm^3^): volume of the buccal defect, obtained by manual segmentation of the defect area in consecutive slices within the VOI, followed by 3D reconstruction and interpolation using the manufacturer’s software (Fig. 3).

**Fig. 4:**
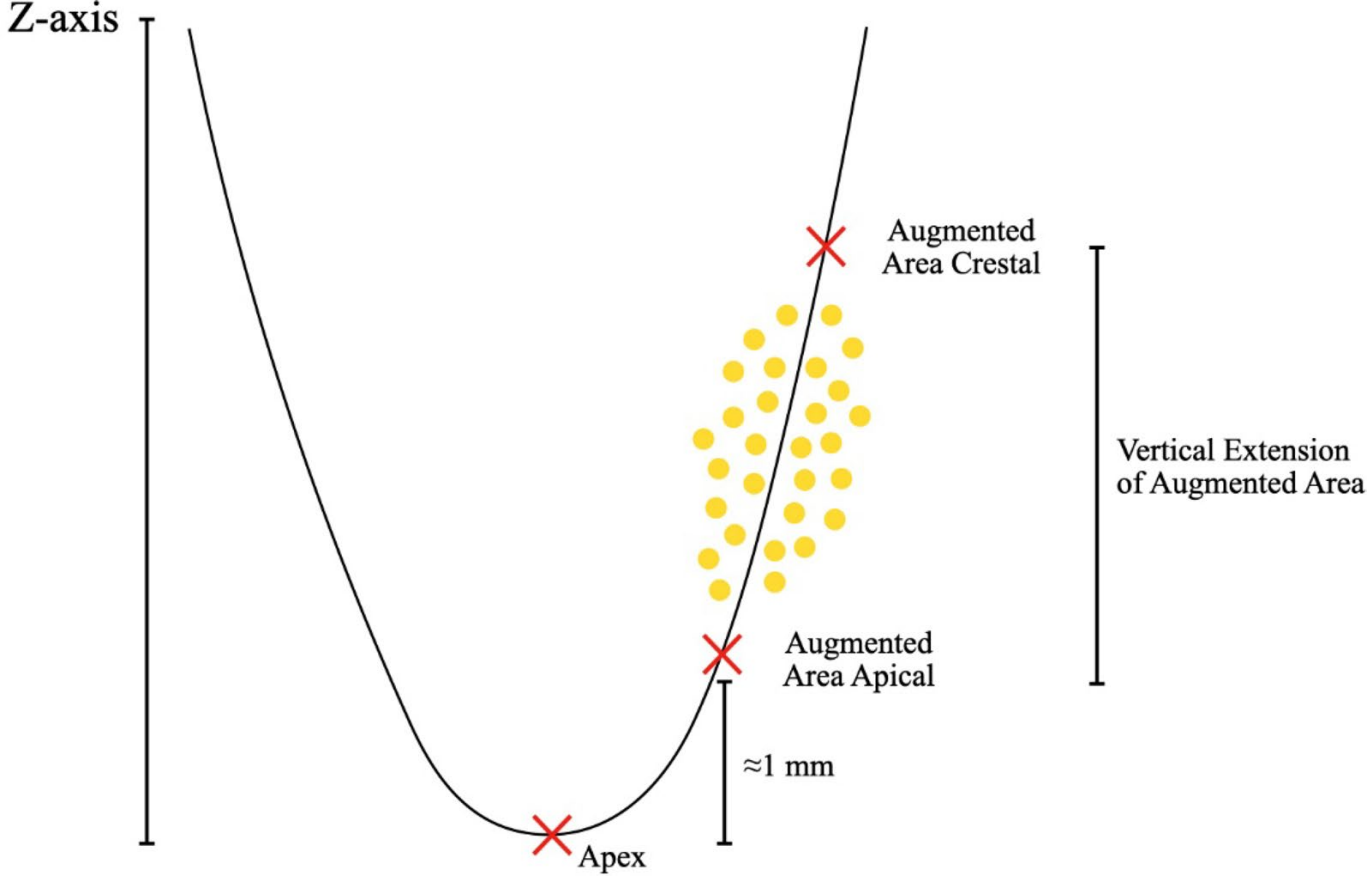
Schematic representation illustrating the determination of horizontal extension at 50% defect height, corresponding to the visible granule height in the micro-CT scan. In the N group, which did not receive any graft material, the 50% reference level was analogously defined based on visible bone remodeling. At this level, linear measurements (e.g., buccal and lingual wall thickness) were performed along the long axis of the mesial root

For the linear measurements, three reference points were defined in each scan (Fig. 4): (i) the apex, (ii) the most coronal crest of the defect, and (iii) the vertical extent of the graft material. From these points, a standardized reference level (50% defect height) was calculated according to the equation: 50% = Apex + 0.5 × (Crestal-Apex).

**Fig. 5:**
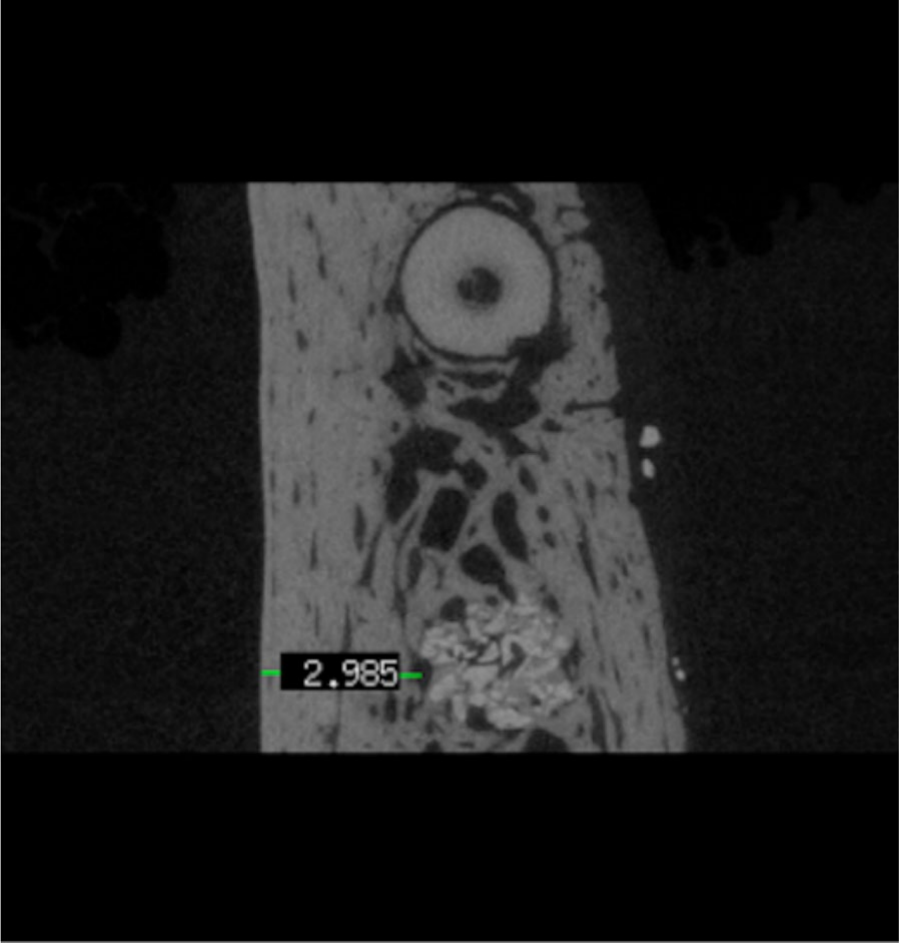
Representative micro-CT image illustrating the measurement of cortical bone thickness at 50% defect height on the lingual aspect

In the grafted groups, (i.e., T1-4 and C groups) the reference point of 50% defect height corresponded to the midpoint between the most apical extent of the graft material and the crestal bone level. In the N group, the 50% defect height was defined analogously, based on visible bone remodeling. The following linear measurements were assessed:

- Horizontal buccal wall thickness (BBW, mm) and lingual wall thickness (LBW, mm, Fig. 5): buccal/lingual cortical bone thickness measured at the 50% defect height.
- Vertical bone height (VBH, mm): distance between the lower border of the orthogonal projection from the tooth axis intercecting with the apex of the tooth visible in the scan. And the deepest location within the (regenerated) defect.

Additionally, the presence of residual graft particles outside the augmented socket was assessed and recorded as 0 (particles confined within the extraction socket) or 1 (particles detected outside the socket).

### Statistical analysis

Statistical analysis was performed using R software (R Development Core Team, 2016). A total of 46 sockets were available for analysis, distributed across the groups as follows: C (n = 7), N (n = 7), T1 (n = 8), T2 (n = 8), T3 (n = 9), and T4 (n = 7). Descriptive statistics for all volumetric and linear parameters (BV/TV, Tb.Th, Tb.Sp, BS/BV, BBD, VBH, BBW, LBW) were presented as median and interquartile range (minimum and maximum). The animal served as statistical unit. Intergroup comparisons were performed using the Kruskal–Wallis test, with p < 0.05 considered statistically significant.

## Results

### Clinical observations

All treated sites healed uneventfully. No soft-tissue dehiscences, infections, swelling, or abscess formation were noted at any of the test and control sites.

### Volumetric assessments

All assessed volumetric and linear measurements are depicted in Table 1.

**Table 1:** Volumetric and linear measurenets assessed in each group (mean, median, minimum and maximum)

Parameter	C	N	T1	T2	T3	T4
*Volumetric measurements*						
BV/TV (%)	76.5	79.8	64.6	68.2	69.0	66.5
Median	74.3	79.7	59.4	67.4	73.2	67.7
Min	69.9	62.5	43.2	46.5	49.1	50.3
Max	87.3	96.7	84.0	88.6	83.7	82.3
Tb.Th (mm)	0.39	0.46	0.38	0.40	0.42	0.41
Median	0.35	0.38	0.37	0.41	0.42	0.41
Min	0.30	0.30	0.29	0.25	0.33	0.28
Max	0.55	0.74	0.48	0.54	0.56	0.52
Tb.Sp (mm)	0.21	0.29	0.41	0.35	0.38	0.41
Median	0.21	0.32	0.42	0.35	0.34	0.42
Min	0.13	0.11	0.11	0.18	0.19	0.25
Max	0.32	0.51	0.68	0.48	0.58	0.52
BS/BV (mm^2^/mm^3^)	6.70	5.38	6.63	6.42	6.20	6.28
Median	6.14	5.98	6.71	6.07	6.29	5.94
Min	4.16	2.63	4.69	3.92	4.07	4.64
Max	8.94	8.30	8.32	9.84	8.84	8.66
BBD (mm^3^)	10.97	18.82	20.08	16.03	11.68	21.73
Median	9.27	8.42	17.62	5.97	12.24	17.93
Min	2.20	0.0	0.67	0.03	0.16	6.74
Max	21.5	50.72	43.85	49.12	20.76	41.32
*Linear measurements*						
VBH (mm)	6.72	6.00	6.63	5.39	6.25	6.30
Median	6.99	6.14	7.03	5.88	6.12	6.53
Min	3.15	5.45	4.31	3.29	4.35	4.50
Max	9.11	6.14	8.51	7.53	7.80	8.31
BBW (mm)	1.31	1.24	1.85	1.57	1.69	1.64
Median	1.11	1.56	1.75	1.56	1.74	1.62
Min	0.97	0.0	1.14	0.73	0.92	1.40
Max	2.07	1.79	3.06	2.24	2.84	2.03
LBW (mm)	2.79	2.38	3.26	2.59	2.97	2.98
Median	2.98	2.37	2.79	2.52	2.81	2.79
Min	2.28	1.16	2.22	1.68	1.90	2.27
Max	3.45	3.60	5.59	4.32	4.32	3.74
Presence of residual graft particles outside the augmented socket (sites%)	5/7 (71.4%)	0	1/7 (12.5%)	1/6 (14.3%)	3/6 (33.3%)	1/6 (14.3%)

The lowest BV/TV values were measured in the T1 group (64.6%) and highest in the N group (79.8%), followed by the C group (76.5%). In the T2, T3, and T4 groups the corresponding values were 68.2%, 69.0%, and 66.5%, respectively. The Tb.Th values among the test groups ranged from 0.38 mm in the T1 group to 0.42 mm in the T3 group. In the C and N groups the emnas Tb.Th values were 0.39 and 0.46 mm, respectively. The Tb.Sp values in the test groups varied between 0.35 mm (T2 group) and 0.41 mm (T1 and T4 groups). Lower values, of 0.21 and 0.29 mm, were obtained in the C and N groups, respectively.

The BS/BV values were similar across the groups, ranging from 6.20 mm^2^/mm^3^ in the T3 group to 6.70 mm^2^/mm^3^ in the C group, with slightly lower values found in the N group (5.38 mm^2^/mm^3^). The largest mean BBD value was found in the T4 group (21.73 mm^3^), followed by the T1 group (20.08 mm^3^), whereas the smallest BBD was detected in the T3 group (11.68 mm^3^). In the C and N groups the respective measurements amounted to 10.97 and 18.82 mm^3^. No statistically significant differences were detected among the groups for any of the volumetric parameters.

### Linear assessments

The VBH assessments in the test groups ranged between 5.39 mm (T1 group) and 6.63 mm (T1 group). In the C and N groups the respective measurements amounted to 6.72 and 6.0 mm.

The highest BBW values were obtained in the T1 group (1.85 mm) and lowest in the N group and the C group (1.24 and 1.31 mm, respectively). The LBW values varied between 2.59 mm (T2 group) and 3.26 mm (T1 group) among the test groups. In the C and N groups, the corresponding measurements were 2.79 and 2.38 mm. No statistically significant differences were detected among the groups for any of the linear measurements.

Particles located outside the socket were most frequently detected in the C group (71.4% of sites), followed by the T3 group (33.3% of sites), and least often in the T1 and T4 groups (12.5% and 12.5% of sites, repsectively). No statistically significant differences were found among groups.

## Discussion

The present preclinical study aimed to evaluate the efficacy of injectable bone fillers for ARP in an established canine model [3, 14]. Based on the micro-CT analysis, the test groups (i.e., injectable bone fillers, T1-4 groups) exhibited slightly lower BV/TV values compared to the C and N groups, which may indicate a slower rate of new bone formation within the extraction sockets. Likewise, the Th.Sp assessments were generally higher in the test groups than in the C and N groups, which could be indicative of a slightly delayed bone remodeling process associated with the injectable materials. On the other hand side, the BBD values tended to be higher in all test groups compared to the C group, suggesting a potentially more favorable maintenance of the ridge dimensions with the non-injectable bone filler. Nonetheless, no statistically significant differences were detected among the groups for any of the volumetric parameters, indicating no major differences in bone regeneration outcomes across all treatment modalities.

Upon further analysis of the present data, after 12 weeks of healing, the linear horizontal measurements on the lingual aspect tended to be higher than those on the buccal aspect across all groups, suggesting a greater dimensional reduction on the buccal side. Overall, all linear measurements appeared were comparable across all investigation groups, with no statistically significant differences detected among them. These findings are consistent with previous histomorphometric observations from an experimental canine study, which reported that between 2 and 8 weeks after tooth extraction, the buccal bone wall was considerably thinner than its lingual counterpart [3]. In the same study, the buccal bone crest was positioned more apically than the lingual counterpart by an average of 0.9 ± 0.3 and 1.9 ± 0.2 mm after 4 and 8 weeks of healing, respectively [3]. The present micro-CT results are also in agreement with the histomorphometric measurements assessed in this experimental study, that were reported in a separatle publication [13]. Specifically, both lingual and buccal bone widths (LBW and BBW) tended to increase from the crestal region (1 mm infracrestally) toward the apex (1 mm and 5 mm infracrestally), with slightly higher values consistently observed on the lingual side compared to the buccal aspect [13].

Nonetheless, in contrast are the results of one recent experimental study in a canine model that used micro-CT to evaluate the dimensional changes occurring after 2 and 8 weeks following ARP employing either a particulated bone mineral or a hydrogel containing gelatin nanoparticles [15]. This study revealed that sites treated with ARP experienced significantly less vertical and horizontal bone loss compared to sites undergoing spontaneous healing regardless of the consistency of the material (i.e., particulated or gel) [15]. The discrepancy noted between the aforementioned studies and the present results may be attributed to differences in the different timepoints for the measurement assessment and methodology used for measuring alveolar ridge dimensions.

One former preclinical study in a canine model demonstrated that the compressive forces exerted on a particulate graft material during ARP directly influence the penetration of the graft material into the apical area of the socket and subsequent new bone formation [12]. Specifically, a particulate synthetic bone filler was applied to post-extraction sockets using forces of 10 g, 50 g, or 200 g, while control sites were left to heal spontaneously [12]. Histomorphometric analysis performed after 8 weeks revealed that graft particles penetrated up to the apical third in the group treated with a force of 200 g, but not in the other test groups or the control sites [12]. Likewise, the percentage of new bone formation was higher in both the coronal and apical thirds of the sockets treated with 200 g compared to those treated with 10 g or 50 g [12]. In light of these findings, one might speculate that the use of an injectable bone filler results in a more homogeneous filling of the extraction sockets, thereby eliminating the application force as a confounding factor influencing the fill of the treated site. In addition, as observed in the present study, residual graft particles outside the augmented socket were less frequently present in the test groups compared to the C group, which may in turn promote more direct bone formation by reducing the interference of non-integrated particles.

In the present study, linear and volumetric measurements were performed employing micro-CT, which, as shown by several former studies, provides significantly correlated data to histomorphometry [16–19]. It should also be acknowledged that to allow for a quantitative analysis, in the present study, test and control treatments were performed in standardized defects, which, however, do not reflect the clinical reality (e.g., non-intact post-extraction sockets) and may have biased the obtained results. Also, the selection and standardization of the VOI, while ensuring comparability, limited the evaluation to a defined region and did not capture the entire augmented socket. Finally, the present study performed the evaluation at a healing time point of 12 weeks. Bone remodeling is a dynamic and time-dependent process, and earlier or later observation periods might have revealed differences in healing kinetics, graft resorption patterns, or dimensional changes that were not detectable at the selected endpoint.

Within its limitations, the present study showed comparable efficacy of injectable bone fillers in maintaining alveolar ridge dimensions compared to C and N.

## Data Availability

No datasets were generated or analysed during the current study.

## References

[1] Tan WL, Wong TL, Wong MC, Lang NP. A systematic review of post-extractional alveolar hard and soft tissue dimensional changes in humans. Clin Oral Implant Res. 2012;23(Suppl 5):1–21. 10.1111/j.1600-0501.2011.02375.x. 10.1111/j.1600-0501.2011.02375.x 22211303

[2] Avila-Ortiz G, Elangovan S, Kramer KW, Blanchette D, Dawson DV. Effect of alveolar ridge preservation after tooth extraction: a systematic review and meta-analysis. J Dent Res. 2014;93(10):950–8. 10.1177/0022034514541127. 24966231 10.1177/0022034514541127 PMC4293706

[3] Araújo MG, Lindhe J. Dimensional ridge alterations following tooth extraction. an experimental study in the dog. J Clin Periodontol. 2005;32(2):212–8. 10.1111/j.1600-051X.2005.00642.x. 15691354 10.1111/j.1600-051X.2005.00642.x

[4] Schropp L, Wenzel A, Kostopoulos L, Karring T. Bone healing and soft tissue contour changes following single-tooth extraction: a clinical and radiographic 12-month prospective study. Int J Periodontics Restor Dent. 2003;23(4):313–23. 12956475

[5] Chappuis V, Engel O, Reyes M, Shahim K, Nolte LP, Buser D. Ridge alterations post-extraction in the esthetic zone: a 3D analysis with CBCT. J Dent Res. 2013;92(12 Suppl):195s–201s. 10.1177/0022034513506713. 24158340 10.1177/0022034513506713 PMC3860068

[6] Hämmerle CH, Araújo MG, Simion M. Evidence-based knowledge on the biology and treatment of extraction sockets. Clin Oral Implant Res. 2012;23(Suppl 5):80–2. 10.1111/j.1600-0501.2011.02370.x. 10.1111/j.1600-0501.2011.02370.x 22211307

[7] Avila-Ortiz G, Chambrone L, Vignoletti F. Effect of alveolar ridge preservation interventions following tooth extraction: a systematic review and meta-analysis. J Clin Periodontol. 2019;46(Suppl 21):195–223. 10.1111/jcpe.13057. 30623987 10.1111/jcpe.13057

[8] Suárez-López Del Amo F, Monje A. Efficacy of biologics for alveolar ridge preservation/reconstruction and implant site development: an American academy of periodontology best evidence systematic review. J Periodontol. 2022;93(12):1827–47. 10.1002/jper.22-0069. 35841608 10.1002/JPER.22-0069 PMC10092438

[9] Atieh MA, Alnaqbi M, Abdunabi F, Lin L, Alsabeeha NHM. Alveolar ridge preservation in extraction sockets of periodontally compromised teeth: a systematic review and meta-analysis. Clin Oral Implant Res. 2022;33(9):869–85. 10.1111/clr.13975. 10.1111/clr.13975 35818637

[10] Apaza-Bedoya K, Magrin GL, Romandini M, Blanco-Carrión J, Benfatti CAM. Efficacy of alveolar ridge preservation with xenografts and resorbable socket sealing materials in the esthetic region: a systematic review with meta-analyses. Clin Implant Dent Relat Res. 2024;26(1):4–14. 10.1111/cid.13257. 37674334 10.1111/cid.13257

[11] Dahl S, Klär-Quarz V, Schulz A, Karl M, Grobecker-Karl T. In vitro handling characteristics of a particulate bone substitute for ridge preservation procedures. Materials (Basel). 2024. 10.3390/ma17020313. 38255481 10.3390/ma17020313 PMC10817230

[12] Delgado-Ruiz R, Romanos GE, Alexandre Gerhke S, Gomez-Moreno G, de Maté-Sánchez Val JE, Calvo-Guirado JL. Biological effects of compressive forces exerted on particulate bone grafts during socket preservation: animal study. Clin Oral Implant Res. 2018;29(7):792–801. 10.1111/clr.12942. 10.1111/clr.12942 27485371

[13] Schwarz F, Ramanauskaite A, Obreja K, Lorenz J, Sader R, Parvini P. Efficacy of injectable bone fillers for alveolar ridge preservation: a histomorphometrical analysis. J Clin Periodontol. 2025;52(7):949–59. 10.1111/jcpe.14162. 40211669 10.1111/jcpe.14162 PMC12176456

[14] Araújo MG, Lindhe J. Ridge preservation with the use of Bio-Oss collagen: a 6 month study in the dog. Clin Oral Implant Res. 2009;20(5):433–40. 10.1111/j.1600-0501.2009.01705.x. 10.1111/j.1600-0501.2009.01705.x 19522974

[15] Yuan S, Li Q, Chen K, et al. Ridge preservation applying a novel hydrogel for early angiogenesis and osteogenesis evaluation: an experimental study in canine. J Biol Eng. 2021;15(1):19. 10.1186/s13036-021-00271-8. 34289877 10.1186/s13036-021-00271-8 PMC8293569

[16] Chappard D, Retailleau-Gaborit N, Legrand E, Baslé MF, Audran M. Comparison insight bone measurements by histomorphometry and microCT. J Bone Miner Res. 2005;20(7):1177–84. 10.1359/jbmr.050205. 15940370 10.1359/JBMR.050205

[17] Fajardo RJ, Ryan TM, Kappelman J. Assessing the accuracy of high-resolution X-ray computed tomography of primate trabecular bone by comparisons with histological sections. Am J Phys Anthropol. 2002;118(1):1–10. 10.1002/ajpa.10086. 11953940 10.1002/ajpa.10086

[18] Müller R, Van Campenhout H, Van Damme B, et al. Morphometric analysis of human bone biopsies: a quantitative structural comparison of histological sections and micro-computed tomography. Bone. 1998;23(1):59–66. 10.1016/s8756-3282(98)00068-4. 9662131 10.1016/s8756-3282(98)00068-4

[19] Park YS, Kim S, Oh SH, et al. Comparison of alveolar ridge preservation methods using three-dimensional micro-computed tomographic analysis and two-dimensional histometric evaluation. Imaging Sci Dent. 2014;44(2):143–8. 10.5624/isd.2014.44.2.143. 24944964 10.5624/isd.2014.44.2.143 PMC4061298

